# Outpatient and Home Chemotherapy with Novel Local Control Strategies in Desmoplastic Small Round Cell Tumor

**DOI:** 10.1155/2008/261589

**Published:** 2008-06-11

**Authors:** Dolly Aguilera, Andrea Hayes-Jordan, Peter Anderson, Shiao Woo, Margaret Pearson, Holly Green

**Affiliations:** ^1^Department of Pediatrics, University of Texas MD Anderson Cancer Center, Houston, TX 77030, USA; ^2^Department of Surgical Oncology, University of Texas MD Anderson Cancer Center, Houston, TX 77030, USA; ^3^Department of Radiation Oncology, University of Texas MD Anderson Cancer Center, Houston, TX 77030, USA

## Abstract

Desmoplastic Small Round Cell Tumor (DSRCT) has a very poor prognosis. This report illustrates novel chemotherapy and local control interventions in a 5-year old patient. The patient was treated in the outpatient setting, achieved remission, with excellent quality of life. The patient presented with massive ascites and >1000 abdominal tumors. Neoadjuvant chemotherapy included vincristine (1.5 mg/m^2^), ifosfamide (3 g/m^2^/day × 3), dexrazoxane/doxorubicin (750/75 mg/m^2^), and etoposide (150 mg/m^2^). Continuous hyperthermic peritoneal perfusion (CHPP) with cisplatin (100 mg/m^2^) was given after extensive cytoreductive surgery. This was followed by irinotecan (10 mg/m^2^/day × 5 × 2 weeks) + temozolomide monthly × 2, then abdominal radiation 30 Gy with simultaneous temozolomide (100 mg/m^2^/day × 5). A total of 12 cycles of irinotecan and temozolamide were given. Except for initial chemotherapy, subsequent courses were in the outpatient setting. Focal retroperitoneal relapse at 18 months was treated with IMRT with bevacizumab (5 mg/kg) and 2 perihepatic metastases with radio frequency ablation/cryoablation followed by chronic outpatient maintenance chemotherapy (valproic acid, cyclophosphamide, and rapamycin). Almost 2 years from diagnosis, the patient maintained an excellent quality of life. This is a novel approach to the treatment of children with massive abdomino-pelvic DSRCT.

## 1. INTRODUCTION

DSRCT is an aggressive neoplasm usually involving numerous peritoneal metastases. It can also occur in different locations including the intrathoracic cavity, pleura, paratesticular, and soft tissues. A specific recurring t(11;22) translocation involving EWS and WT-1 is a characteristic [1]. Since the initial description of DSRCT by Gerald and Rosai [2] in 1991, significant progress has been made in its pathologic identification and diagnosis. However, despite multiple treatment strategies among them, several chemotherapy regimens active for Ewing's sarcoma, aggressive debulking surgery, whole abdominal radiation, high-dose chemotherapy with autologous stem cell transplant, DSRCT survival has not significantly improved. Despite much time in the hospital and morbidity of interventions, durable remissions remain rare. Preliminary data supports the combination of standard treatment modalities with continuous hyperthermic peritoneal perfusion (CHPP) in adult patients with peritoneal carcinomatosis [3–6]. Thus, CHPP may be a rational approach to improve local control of patients with DSRCT [7]. Experience with active chemotherapy agents for DSRCT in the outpatient clinic and also home infusion settings are presented here.

## 2. CASE HISTORY

A 5-year old patient presented to an outside institution with one-week history of abdominal pain and severe constipation not improved with laxatives. Worsening of symptoms and abdominal distention prompted additional evaluation. Abdominal CT scan demonstrated extensive peritoneal masses (6 cm right lower abdomen; 5 cm between rectum and bladder, 8 cm pelvic floor above the bladder), peritoneal and omental implants (estimated number of metastases >1000), and massive ascites. There were many perihepatic lesions, but no obvious tumors within the liver. The patient was referred to MD Anderson Cancer Center, Children's Cancer Hospital. An exploratory laparotomy was done for biopsy and staging purposes; ascites was drained and a temporary peritoneal catheter for ascites removal was placed. The tumor was limited to the abdominal cavity. Other organs had no evidence of disease. Histologic exam of the ascities fluid demonstrated some DSRCT cells in a background of lymphocytosis.

**Figure 1:**
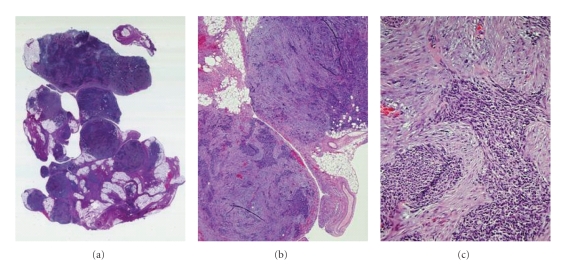
*Peritoneal nodule of DSRCT at diagnosis*. Nodules are composed of fibrocollagenous tissue and irregular islands of malignant undifferentiated small round cells. Immunoperoxidase stains (not shown) in this case were strongly positive for EMA, vimentin, and desmin, partially positive for CD99 and negative for synaptophysin, chromogranin, myogenin, SMA, Bcl-2, CD3, and CD20. Cytogenetic analysis demonstrated t(11;22)(p15;q12). Magnification: (a) × 10, (b) × 40, (c) high power.

Pathology of tumor biopsies (see Figure 1) showed features of a highly anaplastic and somewhat pleomorphic small blue cell malignancy. The immunohistochemical profile included: EMA 4+ in 75% of neoplastic cells with cytoplasmic (granular) pattern; Vimentin: 4+ in 95% of neoplastic cells with cytoplasmic finely and coarsely granular pattern, CD-99/Myc-2 2+ immunoreactivity in 30% of neoplastic cells cytoplasmic and granular pattern, Desmin 4+ in 95% of tumor cells, Myogenin (-), SMA (-), Bcl-2 (-), CD3 (-), CD20 (-), Synaptophysin (-), Chromogranin (-). Cytogenetic analysis confirmed the presence of t(11;22)(p15;q12)by FISH characteristic of DSRCT.

### 2.1. Preadjuvant chemotherapy treatment

**Figure 2:**
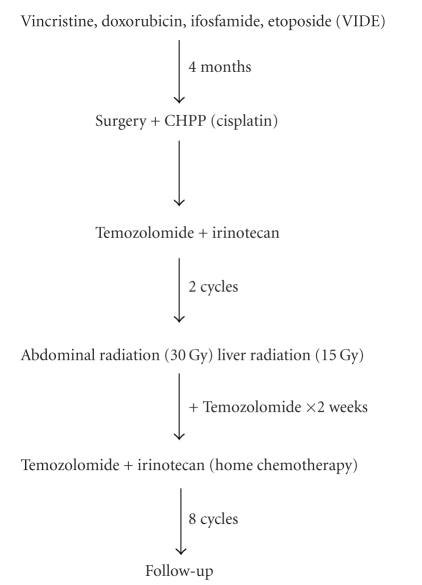
*Outpatient neoadjuvant and adjuvant chemotherapy plan for DSRCT*. After initial diagnosis and treatment of peritoneal, abdominal and pelvic disease, systemic chemotherapy agents were administered on an outpatient basis followed by local control with cytoreductive surgery + CHPP with cisplatin, then whole abdomen radiation with temozolomide as a radiation sensitizer, followed by temozolomide and irinotecan. Outpatient therapy was 15 months.

Figure 2 shows a sequence of Neoadjuvant and adjuvant chemotherapy. First cycle was vincristine (1.5 mg/m^2^), cyclophosphamide (2.2 g/m^2^), and doxorubicin (75 mg/m^2^) as an inpatient. Subsequent chemotherapy was outpatient and used a modified “VIDE” regimen similar to EuroEwings protocols every three weeks in 4 cycles. Vincristine 1.5 mg/m^2^ was followed by dexrazoxane (750 mg/m^2^ IV over 15 minutes) + doxorubicin (75 mg/m^2^ IV over 15 minutes) and etoposide (150 mg/m^2^ IV over 1 hour). Ifosfamide (3 g/m^2^) was mixed with MESNA (3 g/m^2^/day) in volume of 240 mL normal saline and given as a continuous infusion × 3 days (total dose 9 g/m^2^) followed by 1 day of MESNA (3 g/m^2^). Pegfilgrastim was given after completion of MESNA when port was deaccessed by the home health nurse.

Day 1 chemotherapy was given in the outpatient clinic; ifosfamide infusions were done at home with bag changes by home health nursing. The patient had excellent performance and attended elementary school. Just prior to local control surgery, an additional cycle consisting of vincristine and liposomal doxorubicin (40 mg/m^2^ IV over 3 hours with hands and feet on ice to reduce risk of palmar/plantar erythrodysesthesia) was administered. Total anthracycline dose was 415 mg/m^2^.

### 2.2. Local control phase of therapy: CHPP and radiation

Five months from initial presentation, and after approval by the institutional review board, the patient underwent aggressive cytoreductive surgery including greater and lesser omentectomy, cholecystectomy and partial peritonectomy and removal of 402 metastatic nodules.

**Figure 3:**
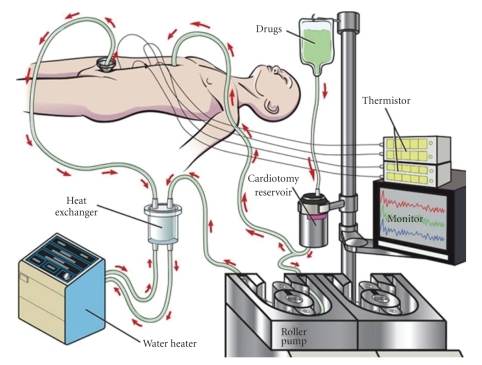
*Continuous hyperthermic peritoneal perfusion (CHPP)*. The CHPP procedure involves placing temperature probes, inflow and outflow cannulas, and continuous perfusion of plasmalyte followed by cisplatin containing chemotherapy as the perfusate at 41-42°C. To achieve this, the inflow was usually about 45°C. Manual agitation of the closed abdomen was done for 1.5 hours to ensure that all peritoneal surfaces were exposed to the cisplatin.

At the end of the surgical procedure, cisplatin 100 mg/m^2^ at 40–42°C was continuously perfused in the peritoneal cavity. CHPP (Figure 3) consisted of the placement of 7 temperature probes: (1) ligament of Treitz, (2) right lobe of the liver, (3) right upper quadrant, (4) left upper quadrant, (5) right lower quadrant, (6) left lower quadrant, and (7) sigmoid colon mesentery. The abdomen was then closed temporarily to secure the inflow and outflow cannulas. A Y-shaped inflow cannula was placed above the liver, and a pelvic drain was placed in the pelvis.

Plasmalyte was perfused for 15 to 20 minutes to establish equivalent temperatures. When average temperatures of 40°C were established in all portions of the abdomen,cisplatin 100 mg/m^2^ was added to the perfusate simultaneous with the beginning of an intravenous infusion of sodium disulfate. The abdomen was continuously agitated for 90 minutes, temperatures were monitored frequently, and agitation was adjusted according to the temperature of each probe. The temperature of the inflow and the thermomistor was 45°C. Temperature in the intra-abdominal probes was about 41-42°C. The volume was 2.5 liters. Flow rate was 700 mL/minute throughout the 1.5 hour CHPP procedure. Cisplatin was drained, and then two liters of Plasmalyte at room temperature were infused to rinse cisplatin from the peritoneal cavity. Abdomen was reopened and inspected. Gastrostomy and jejunostomy tubes were placed in anticipation of enteral nutrition in the post-operatory period. The patient had a rapid recovery and was discharged from the hospital to home 7 days after surgery.

Pathology evaluation from peritoneal tumor nodules reported minimal residual malignant small tumor cells in most of the specimens consistent with microscopic residual disease.

### 2.3. Adjuvant DSRCT chemotherapy and radiosensitization program

Outpatient and home chemotherapy were provided before and during radiation (6 months from diagnosis) and then for an additional 8 cycles until 14 months after diagnosis. Regimen was similar to that reported by Wagner et al. [8]. Temozolamide (100 mg/m^2^/day × 5 days) and irinotecan (10 mg/m^2^/day × 5 days × 2 weeks) for 2 four-week cycles. During total abdominal radiation (3 Gy × 10 fractions; total 30 Gy and 15 Gy to the liver/kidneys) irinotecan was not given and only temozolomide (100 mg/m^2^/dose × 5 days × 2 cycles) was provided. After radiation, 8 cycles of temozolomide + irinotecan were given. Day 1: temozolomide and irinotecan were given in the outpatient clinic each month. Days 2–5 and 8–12: irinotecan chemotherapy infusions were done at home (after school) using home health nursing.

The patient tolerated this chemotherapy well and had one, brief hospitalization for fever and neutropenia. He was able to attend elementary school with almost perfect attendance.

### 2.4. Relapse: additional local control with IMRT, then radio frequency ablation + cryoablation

**Figure 4:**
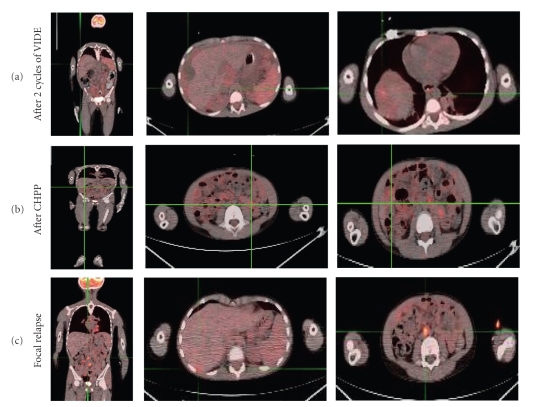
*PET-CT fusion imaging*. (a) Residual uptake in the peritoneal implants after 2 cycles of therapy, SUV 3. (b) No evidence of FDG avid disease after cytoreductive therapy. (c) Relapse in the perihepatic space including dome of the liver (SUV 2.8) and retroperitoneal nodes (SUV 5).

Although the patient continued to be healthy, attend school, and had no symptoms, routine PET-CT done 2 months after completion of therapy showed five FDG avid areas (Figure 4). All were just outside of the peritoneum and at edge or outside prior radiation fields: two adjacent posterior perihepatic, one at the dome of the right diaphragm, and 2 retroperitoneal pericaval nodes. IMRT (1.5 Gy in 15 fractions; total 45 Gy) was given to the retroperitoneal disease. Radiosensitization was with temozolomide (75 mg/m^2^/day × 5 days × 2 weeks), bevacizumab (5 mg/kg every 2 weeks × 3 doses), valproate (500 mg/m^2^/day divided BID), and rapamycin (2 mg/m^2^/day). The patient tolerated radiotherapy well and had no side effects.

**Figure 5:**
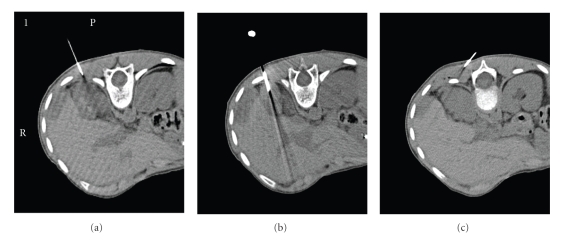
*Radio frequency ablation and cryoablation procedure*. Under direct CT fluoroscopic guidance, an RFA electrode was advanced into the perihepatic lesions. These were heated from 42°C to 43°C for 30 minutes (a) and (b) then a cryoablation probe was advanced and 2 cycles of freeze and thaw for 8–10 minutes (c).

He was reimaged and the adjacent perihepatic lesions had increased in size from 3.0 × 1.5 cm to approximately 3.8 × 2.6 cm and from 3.3 × 1.3 cm to 4.3 × 2.2 cm; there was no change in size of the 1 cm dome of right diaphragm lesion. To destroy cells in the perihepatic nodules and increase chance of mounting an immune response, both radio frequency ablation and cryoablation were used (Figure 5). Under direct CT fluoroscopic guidance, a radio frequency ablation (RFA) electrode (RITA system) was used to heat the tumor to 42-43°C for 30 minutes, then each lesion was frozen using a cryoablation probe (PERC-17; Endocare) for 2 cycles of freezing (5–10 minutes) and thawing (5–8 minutes). The patient tolerated the procedure well and was observed in the hospital overnight. He had no side effects from the procedure except for some skin reaction and scab which resolved in 2 weeks.

### 2.5. Chronic adjuvant chemotherapy program and followup

**Figure 6:**
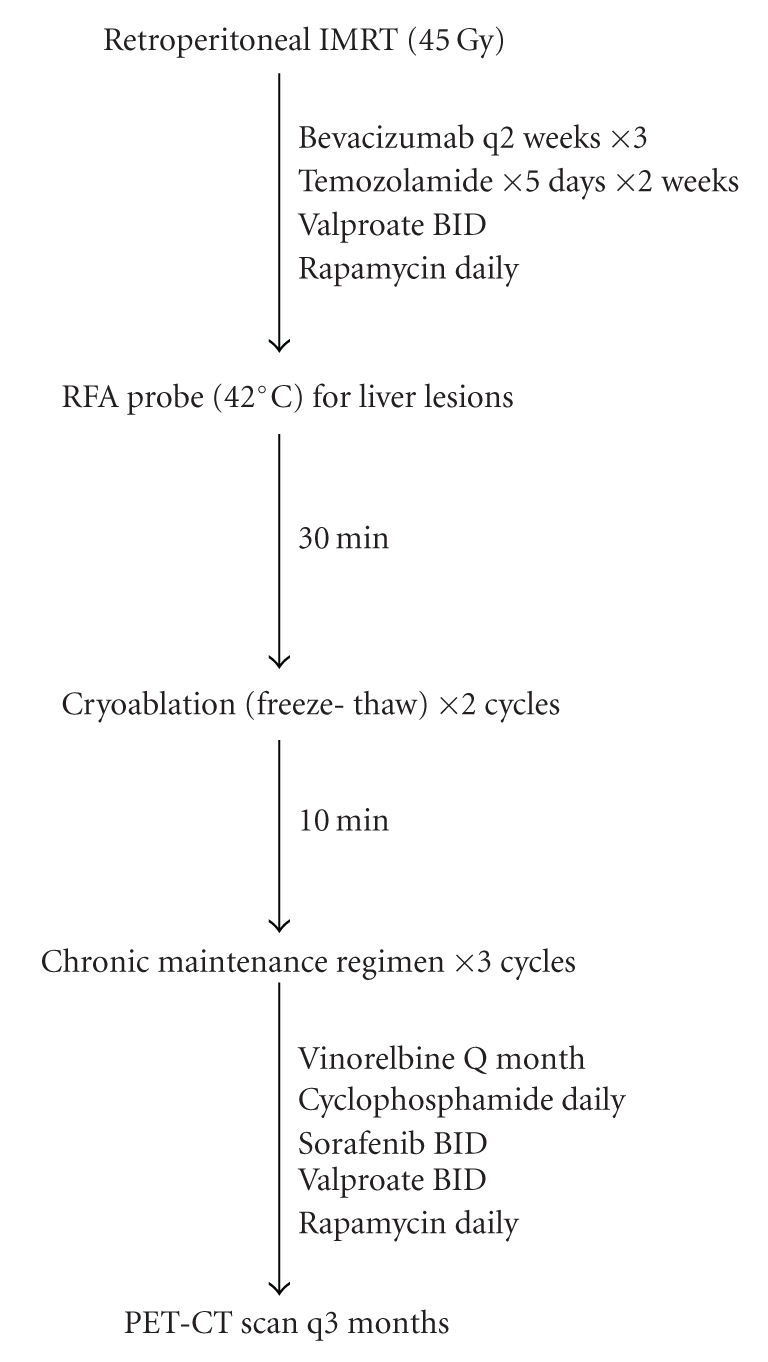
*Schema for local control of relapse foci and continuation adjuvant therapy*. Radiation in combination with bevacizumab was used to pericaval disease. Localized perihepatic lesions were treated with a combination of RFA and cryoablation. These measures were followed with a chronic adjuvant regimen.

The patient received a regimen of valproate (250 mg BID), rapamycin (2 mg daily), and sorafenib (200 mg BID) and chemotherapy similar to that reported by Casanova et al. [9], vinorelbine (25 mg/m^2^ IV monthly) and oral cyclophosphamide (25 mg daily) (see Figure 6). Sorafenib and vinorelbine were discontinued because of significant diarrhea (see treatment schema in Figure 6). The patient attended school full time without symptoms.

For almost 2 years from diagnosis of massive disease at DSRCT, this patient has had only 32 days of hospitalization including initial admission of 18 days, 3 days for hydration, 1 episode for hematuria, 2 days for fever and neutropenia, 7 days for CHPP surgery, and 1 overnight observation for RFA + cryoablation. He will continue on a maintenance chemotherapy regimen and be followed with PET-CT fusion imaging every 3 months.

## 3. RESULTS

### 3.1. Effective chemotherapy as an outpatient

Once ascites improved after initial chemotherapy cycle, subsequent cycles were given in the outpatient clinic. For 13 out of 14 cycles, only day 1 was given at the major medical center, days 2–4 ifosfamide or 2–12 irinotecan were given at home using home infusion services.

Response to initial chemotherapy (Table 1) was evaluated with abdominal CT scans interpreted as a very good partial response (Figure 4), more than 50% reduction of the perihepatic, pelvic, and omental implants when compared with initial CT imaging studies. After one cycle of chemotherapy, PET scan demonstrated diffuse nodules and less ascites throughout the abdomen. PET-CT demonstrated low-grade FDG activity (maximal SUV 3). After completion of 4 months of VIDE, PET scan continued to demonstrate low-FDG uptake in the perihepatic implant with maximum SUV measured at 3 (stable disease). Other nodules did not show significant uptake probably because of small size (<1 cm). PET scan after cytoreductive surgery, CHPP, and one cycle of temozolamide /irinotecan showed residual activity over the dome of the right lobe of the liver may be post treatment-related or post-surgical in nature. After completion of abdominal radiation and temozolomide with irinotecan (6 cycles), there was no evidence of FDG avid disease. This was considered a clinical complete remission.

### 3.2. Excellent quality of life during outpatient and home chemotherapy program

Except for the initial chemotherapy, the treatment regimen for this patient was in the outpatient or home setting, allowing him to have excellent quality of life. This allowed school attendance and play at home. Generally home ifosfamide bag changes or irinotecan infusions could be done after school.

### 3.3. Approach to high-risk status: surveillance detects recurrence before symptoms

Because the patient had a high risk of recurrence, based on the initial presentation with massive ascities and very large number of peritoneal implants, close monitoring for relapse was necessary with PET-CT imaging fusion every 12 weeks during and after therapy. This resulted in detection of recurrence before appearance of clinical symptoms. The PET-CT also provided information useful in IMRT treatment planning.

Chronic adjuvant chemotherapy: well tolerated.

The current therapy designed to treat an Ewing's family of tumor using agents compatible with outpatient or home therapy that could be given for months to years. Because of high-risk status, a long term therapy is planned. The toxicity has been with minimal with two episodes of fever and neutropenia or illness associated with chemotherapy administration to date.

## 4. DISCUSSION

In this report, we have shown a child with high-risk DSRCT with massive disseminated peritoneal DSRCT implants who had complete response and sustained a remission for 21 months following therapy with a carefully designed multimodality outpatient and home treatment plan.

DRSCT has a 15% overall survival at 5 years [10]. Patients presenting with abdominal disease typically are in an advanced stage, with large masses and/or extensive seeding in the visceral and parietal peritoneal layers. Thus, DSRCT in the abdomen is almost always disseminated regionally. The presence of the translocation t(11 : 22) (p13 : q12) involving fusion of the Ewing sarcoma gene (EWS) and the Wilm's tumor gene (WT1) provides the confirmation of diagnosis [1, 11].

Chemotherapy agents with known activity in DSRCT are very similar to those active in Ewing Sarcoma. Both tumors share an EWS fusion protein and may also share molecular mechanisms facilitating proliferation and survival pathways. Alkalyting agents such as cyclophosphamide and ifosfamide are important components of therapy. Currently, a well-recognized treatment schema has been reported by Kushner et al. [12] who described results in 12 DSRCT patients. This intensive alkylator-based therapy used cyclophosphamide, doxorubicin, vincristine alternating with ifosfamide and etoposide. Its combination with other treatment modalities such as surgery, radiation, autologous stem cell rescue, or the combination of all of the above was used. The median survival time was 19 months. For those achieving complete response, the median followup in this series was 22 months, about the same as our patient. The toxicity for this regimen can be substantial and often includes hospitalization not only for chemotherapy, but also fevers associated with myelosupression and mucositis. Our outpatient and home program had significantly less hospitalization and very tolerable side effects. It permitted routine school attendance; play activities were possible with an excellent quality of life.

In our patient, ifosfamide/mesna as a continuous infusion as previously described by Skubitz et al. [13], was well tolerated and allowed treatment in the outpatient setting. No hydration is required. Since a small volume (e.g., 240–360 mL/day) can be infused, this permits a normal pattern of sleep. Irinotecan activity in sarcoma has been shown by Bisogno et al. [14] and Wagner et al. [8]. We also have observed high activity of the temozolomide + irinotecan combination in relapsed Ewing's sarcoma and 2 previous patients with DSRCT [15]. Rossof and Bayliff described successful responses in DSRCT cases to irinotecan [16]. The toxicity of temozolomide and irinotecan was very low. Our patient did not experienced documented infections; home infusions of irinotecan were routine and done after school.

Epigenetic alterations of DSRCT are probable since EWS is associated with responses to such agents. For example, the effects of histone deacetylase (HDAC) inhibitors in Ewing's family tumors was recently described by Sakimura et al. [17]. This group showed expression of the EWS-Fli1 fusion RNA and fusion protein was inhibited with desipeptide. Recent preliminary work shows that both HDAC and mTOR inhibitors are promising not only for EWS, but also DSRCT [18–20]. Low dose of cyclophosphamide has activity in pediatric sarcoma [9]. We have used this information in the design of a chronic, well-tolerated outpatient adjuvant therapy in our DSRCT patient.

Immuno-histochemistry studies revealed in several intra-abdominal DSRCT specimens, upregulation in tumor cells of platelet derived growth factor AB chain and platelet derived growth factor alpha receptor [21]. Perhaps these findings support additional contributions towards tumor survival and/or apoptosis resistance. Thus sorafenib, a multikinase inhibitor that directly inhibits the autophosphorylation of several receptor tyrosine kinases including PDGFR beta, and c-kit may have additional activity beyond VEGF receptor inhibition [22].

Local control is important in DSRCT [10]. In our patient local control measures included surgery with CHPP [5–7, 23], abdominal radiation with radiosensitizing chemotherapy during the first cycle (temozolamide) and with bevacizumab during radiation for relapse [24]. The initial role of surgery in patients with extensive intra-abdominal disease is often limited to biopsies; after chemotherapy response this is followed by a debulking procedure. Saab et al. [25]reported a series of patients with abdominopelvic DRSCT with incomplete resection, in which total surgical resection was not feasible; outcome was poor.

Aggressive cytoreductive surgery is currently accepted to have a primary role in the achievement of prolonged survival of some malignancies involving the peritoneum [4, 6]. In DSRCT Lal reported 3-year OS was 58% in gross total resection in comparison with 0% in the nonresection cohort [10]. Other therapeutic modalities such as CHPP have been found to significantly improve outcome in cancer involving the peritoneum [6, 23, 26–28]. Although Gil et al. [29] showed no benefit in DSRCT and had more toxicity with intraperitoneal cisplatin and doxorubicin, no further multiagent chemotherapy was used in the postoperative period. Our patient was discharged from the hospital on post-op day 7 and subsequently began cycles of adjuvant temozolomide + irinotecan, a non-cross-resistant regimen.

The combination of CHPP and radiotherapy would be expected to result in scarring and adhesions and limit ability to do second-look surgery. Thus the local control option for isolated recurrent perihepatic lesions was radio frequency ablation and cryoablation (Figure 5). The use of this technique in pediatric patients with metastatic DSRCT has been previously reported [30]. Heating increases the expression of heat shock proteins in hepatocellular carcinoma; these molecular chaperones are important for antigen presentation [31]. Cryoablation is a technique to not only kill tumor cells, but avoid protein denaturation by RFA. This approach using heat (RFA probe), then cryoablation can be considered an “in vivo vaccine” and was first developed by S Markovic at Mayo Clinic; a clinical trial is currently underway to study effectiveness in melanoma hepatic metastases.

We found that PET-CT imaging was useful for the evaluation of the extension of disease at presentation as well as a useful tool for monitoring the response to therapy; therefore, we performed PET-CT scans in our patient at every 3 months interval. The use of PET for DSRCT has been described previously by Rosoff and Bayliff [16]. This is the first report of its use in children after the use of continuous peritoneal perfusion and to monitor for recurrence. Reaching a no evidence of disease (NED) status and excellent quality of life was possible in our patient with very extensive abdominopelvic DSRCT using outpatient therapy. Principles and techniques should be applicable to other high-risk young patients with DSRCT.

Finally, because inherent and seemingly inevitable chemotherapy resistance of DSRCT, in addition to efforts at best local control, novel therapeutic ideas are needed in the event of a relapse. The options include new agents such as small molecule inhibitor of C-kit and PDGFR, imatinib mesylate which has a remarkable safety profile. The current experience of the use of imatinib in DSRCT has been recently reported by Bond et al., where no objective response was seen at the dose and schedule tested [32]. Other molecules include IGF-R1 antibodies that inhibit downstream signaling of both the mTOR and MAP kinase pathways that are important in both proliferation and resistance to apoptosis of a variety of cancers. It is noteworthy that patients with Ewing's family of tumors responded in phase I trial of R1507. The SARC consortium phase II trial of R1507 opened in early 2008 (SARC 011; sarctrials.com) and should provide a determine whether this agent is active in DSRCT in ages 12 and up [33]. The safety profile of a multiple ascending dose study for R1507 for children ages 2 to 17 with advanced solid tumors (http://www.clinicaltrials.gov/) is expected to allow participation of younger cohorts (ages 2 and up) on SARC 011 in 2008.

## Figures and Tables

**Table 1:** Outpatient DSRCT chemotherapy: doses and schedule.

Agent	Dose	Route	Comments
Preadjuvant phase of therapy (every 3 weeks × 4 cycles)			

Vincristine	1.5 mg/m^2^	iv over 2–15 minutes	Max dose 2 mg; day 1 only
Etoposide	150 mg/m^2^	iv over 1 hour	Day 1 only
Dexrazoxane	750 mg/m^2^	iv over 15 minutes	Just before doxorubicin
Doxorubicin	75 mg/m^2^	iv over 15 minutes	Day 1 only
Ifosfamide	3 g/m^2^	iv daily × 3	Continuous infusion. Mix
+ MESNA	3 g/m^2^	iv daily × 4	with MESNA in 240–360 mL NS days 1, 2, 3.
			MESNA only day 4 (portable pump as outpatient)

CHPP phase			

Cisplatin	100 mg/m^2^	Intraperitoneal	As continuous peritoneal perfusion at 42°C × 1.5 hr

Radio sensitization during RT			

Temozolomide	75 mg/m^2^	po	Daily × 5 × 2 weeks
Bevacizumab	5 mg/kg	iv	q2 weeks × 2 doses begin before RT

Pre-RT (2 cycles) and post-RT (10 cycles)			

Temozolomide	100 mg/m^2^	po	Daily × 5 each month
Irinotecan	10 mg/m^2^	iv	Daily × 5 × 2 weeks each month; may do as home infusions

Chronic continuation chemotherapy			

Valproate	500 mg/m^2^	po	Daily; divide BID monitor level <100
Rapamycin	2 mg/m^2^	po	Daily round to nearest mg
Cyclophosphamide	25 mg	po	If >1.5 m^2^ may give 50 mg daily. Monitor cbc
